# Empagliflozin and left atrial function in patients with type 2 diabetes mellitus and coronary artery disease: insight from the EMPA-HEART CardioLink‐6 randomized clinical trial

**DOI:** 10.1186/s12933-024-02344-6

**Published:** 2024-08-28

**Authors:** Marina Pourafkari, Kim A. Connelly, Subodh Verma, C. David Mazer, Hwee Teoh, Adrian Quan, Shaun G. Goodman, Archana Rai, Ming Yen Ng, Djeven P. Deva, Piero Triverio, Laura Jiminez-Juan, Andrew T. Yan, Yin Ge

**Affiliations:** 1 Department of Medical Imaging, St. Michael’s Hospital, Toronto, Canada; 2 Division of Cardiology, Terrence Donnelly Heart Centre, St Michael’s Hospital, 30 Bond Street, Toronto, ON M5B 1W8 Canada; 3 University of Toronto, Toronto, Canada; 4 Keenan Research Centre, Li Ka Shing Knowledge Institute, St Michael’s Hospital, Toronto, Canada; 5 Division of Cardiac Surgery, St Michael’s Hospital, Toronto, Canada; 6 Department of Anesthesia, St Michael’s Hospital, Toronto, Canada; 7 Division of Endocrinology and Metabolism, St Michael’s Hospital, Toronto, Canada; 8 Department of Diagnostic Radiology, Li Ka Shing Faculty of Medicine, University of Hong Kong, Hong Kong, China; 9 Department of Electrical & Computer Engineering, Institute of Biomedical Engineering, University of Toronto, Toronto, Canada

**Keywords:** Empagliflozin, Diabetes, Left atrial function, Cardiac MRI

## Abstract

**Background:**

Sodium-glucose cotransporter-2 (SGLT2) inhibitors have demonstrated reduction in heart failure outcomes in patients with type 2 diabetes mellitus, although the exact mechanism of benefit remains unclear. Alteration in left atrial (LA) function due to chronic pressure or volume overload is a hallmark of heart failure.

**Objective:**

To evaluate the effect of the SGLT2 inhibitor empagliflozin on LA volume and function.

**Methods:**

90 patients with coronary artery disease and type 2 diabetes (T2DM) were randomized to empagliflozin (*n* = 44) or placebo (*n* = 46), and underwent cardiac magnetic resonance (CMR) imaging at baseline and after 6 months. The main outcome was change in LA volume; LA function, including active and passive components, was also measured by a blinded reader.

**Results:**

At baseline, there was no significant difference in LA volumes between the empagliflozin (indexed maximum LA volume 26.4 ± 8.4mL/m^2^, minimum LA volume 11.1 ± 5.7mL/m^2^) and placebo (indexed maximum LA volume 28.7 ± 8.2mL/m^2^, minimum LA volume 12.6 ± 5.0mL/m^2^) groups. After 6 months, changes in LA volumes did not differ with adjusted difference (empagliflozin minus placebo): 0.99 mL/m^2^ (95% CI: -1.7 to 3.7 mL/m^2^; *p* = 0.47) for indexed maximum LA volume, and 0.87 mL/m^2^ (95% CI: -0.9 to 2.6 mL/m^2^; *p* = 0.32) for indexed minimum LA volume. Changes in total LA emptying fraction were also similar, with between-group adjusted mean difference − 0.01 (95% CI: -0.05 to 0.03, *p* = 0.59).

**Conclusion:**

SGLT2 inhibition with empagliflozin for 6 months did not have a significant impact on LA volume and function in patients with T2DM and coronary artery disease. (Effects of Empagliflozin on Cardiac Structure in Patients with Type 2 Diabetes [EMPA-HEART]; NCT02998970).

## Introduction

Type 2 diabetes mellitus (T2DM) and its most severe complications—cardiovascular and kidney disease—represent some of the greatest global pandemics of chronic disease today [1]. Sodium-glucose cotransporter-2 (SGLT2) inhibitors have become a mainstay pharmacologic treatment of T2DM due to their demonstrated reduction in heart failure outcomes [2], and progression of chronic kidney disease [3], although the exact mechanism of cardiovascular benefit remains debated [4]. Cardiac magnetic resonance imaging (CMR), considered the gold standard for quantification and characterization of myocardial tissue and function, is well poised to help elucidate the direct cardiac effects of SGLT2 inhibitors. Previous studies have demonstrated that in patients with T2DM and coronary artery disease (CAD), empagliflozin led to reduction in left ventricular (LV) mass [5] and a decrease in extracellular volume (ECV), a measure of diffuse myocardial fibrosis [6].

Under physiologic conditions, left atrial (LA) function contributes to 20–30% of the LV stroke volume, and alteration in its function due to chronic pressure or volume overload is a hallmark of heart failure [7]. Alteration in LA function, with increased chamber volume and decreased compliance, has been demonstrated in experimental models of subclinical heart failure with preserved ejection fraction (HFpEF) [8]. Importantly, LA size and function have demonstrated prognostic significance not only in individuals with established cardiovascular disease, but also in a general ambulatory population [9]. However, the impact of SGLT2 inhibition on LA size and function have not been previously described in patients with T2DM and CAD. Accordingly, in this post-hoc sub-study of the EMPA-HEART (Effects of Empagliflozin on Cardiac Structure in Patients with Type 2 Diabetes) CardioLink-6 randomized control trial, we investigated the impact of SGLT2 inhibition with empagliflozin on parameters of LA size and function, and their relationship with natriuretic peptide and measures of LV diastolic function.

## Materials and methods

The design and main results of the EMPA-HEART CardioLink-6 trial (NCT02998970) have been published [5]. Briefly, EMPA-HEART CardioLink-6 was a double-blinded randomized controlled trial, which between November 2016 and April 2018, recruited individuals ≥ 40 and ≤ 80 years old with glycated hemoglobin (HbA1c) between 6.5 and 10.0%, known CAD, and estimated glomerular filtration rate ≥ 60 mL/min/1.73^2^. Key exclusion criteria included coronary revascularization within the past 2 months, LV ejection fraction (LVEF) < 30%, New York Heart Association IV symptoms of heart failure or hospitalization for heart failure in the past 3 months. Participants provided informed consent and were randomized (1:1) to empagliflozin 10 mg or placebo daily for 6 months.

Clinical, echocardiography, and CMR characteristics were collected at the baseline visit, where blood samples were also drawn. Three clinical visits were performed over the six-month follow-up period. At the final visit, CMR was repeated. Plasma N-terminal pro b-type natriuretic peptide (NT-proBNP) levels were quantified on the day of collection using the Cobas 6000 e601 immunology analyzer (Roche Diagnostics, Mississauga, ON, Canada).

### Acquisition and analysis of CMR images

CMR examinations were performed using a 3T MRI scanner (MAGNETOM Skyra; Siemens Healthcare, Erlangen, Germany). Segmented balanced steady-state free-precession (bSSFP) sequences were used for standard cine CMR acquisition with retrospective ECG-gating. 3 long-axis views and a stack of short-axis slices covering the entire heart were acquired with typical parameters, as previously described. 10 min post intravenous contrast administration (0.1 mmol/kg of gadolinium chelate [Gadovist]; Bayer Schering Health Care Limited, Reading, United Kingdom), late gadolinium enhancement (LGE) images were acquired in matching long-axis and short-axis slices.

CMR analyses were performed using a commercially available software (cvi42, Circle Cardiovascular Imaging Inc, Calgary, AB, Canada). Manual tracing of the epicardial and endocardial contours at end-diastole and end-systole were used to determine the left and right ventricular volumes. LV mass was calculated using the LV myocardial volume using the summed contiguous short-axis slices, multiplied by myocardial density.

LA measurements were performed by a single, blinded, operator (MP). Manual adjustments of automated contours were performed in the long-axis 4- and 2- chamber view at each of the cardiac phase. The LA appendage and pulmonary veins were excluded due to anatomic variability between patients and to preserve reproducibility.

**Fig. 1:**
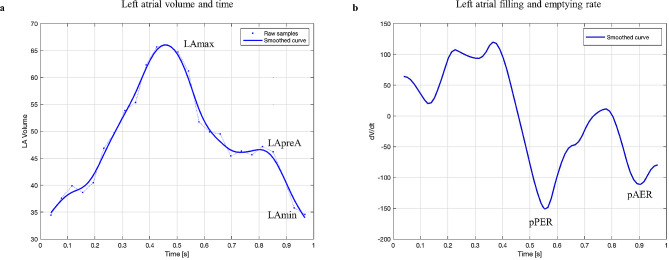
Conceptual diagram demonstrating **a** left atrial volume– time curve throughout the cardiac cycle, with associated maximum, minimum, and pre atrial kick volumes, **b** left atrial filling and emptying rate curve with early (passive) and late (active) peak emptying rates * LAmax* Maximum LA volume, *LAmin* Minimum LA volume, *LApreA* Left atrial volume pre-atrial contraction, *pPER* Peak passive emptying rate, *pAER* Peak active emptying rate

LA volume was calculated using the biplane area-length method, using the formula V = 8(A2)(A4)/3πL, where A2 and A4 represent the LA areas in the 2- and 4-chamber views, and L is the average LA length measured from the midpoint of the mitral annulus to the posterior aspect of the LA [10]. LA function was assessed using the following: LA total emptying fraction (LAEF) = Maximum LA volume (LAmax) – Minimum LA volume (LAmin) / LAmax, LA passive emptying fraction (LAPEF) = LAmax – LA volume pre-atrial contraction (LApreA) / LA max, and LA active emptying fraction (LAAEF)= (LApreA – LAmin) / LApreA (Fig. 1). The LA filling profile curves were then computed in MATLAB (MathWorks, Natick, MA) using a smoothing spline to reduce the effect of acquisition noise and used to generate volume-time curves and their first derivative, allowing for the assessment of early and late LA peak emptying rate. All measurements were performed by an investigator (MP) blinded to clinical data, as well as to treatment allocation, and timing of each scan. Volumes were indexed to baseline body surface area.

## Statistics

Continuous variables are presented as mean ± standard deviation or median (interquartile range) depending on their distribution, while categorical variables are presented as count or percentages. These were compared using either student’s t test or Mann-Whitney U test, and χ^2^ test, respectively. Spearman’s correlation was used to assess the relationships between LA function and LV parameters, blood pressure, and NT-pro-BNP. Changes in LA indices between baseline and 6 months treatment were compared between empagliflozin and placebo groups using ANCOVA and adjusted for baseline values. We tested for interaction between treatment assignment and levels of NT-proBNP, as well as the presence of regional wall motion abnormalities at baseline. Intra-observer reproducibility was evaluated by intra-class correlation coefficient. All analyses were performed using SPSS 25 (IBM, Armonk, NY, USA) and statistical significance was set at a two-sided p value < 0.05.

## Results

Ninety-seven participants were enrolled in the EMPA-HEART CardioLink-6 trial, with 49 randomized to empagliflozin and 48 to placebo, respectively. As 7 participants (5 empagliflozin, 2 placebo) did not complete their six months follow-up, 90 participants formed the cohort for this study.

Baseline demographic, clinical, and imaging characteristics of the participants are summarized in Table 1. Baseline LV mass indexed to body surface area (LVMi) were 59.3 ± 10.9g/m^2^ and 62.2 ± 12.8g/m^2^, in the empagliflozin and placebo groups, respectively. The change in LVMi from baseline to 6 months was − 2.6 ± 7.8g/m^2^ for the empagliflozin group and − 0.01 ± 5.7g/m^2^ for the placebo group, with an adjusted between group difference of – 3.35g/m^2^ (95% CI – 5.9 to − 0.81 g/m^2^, p = 0.01). The mean change in HbA1c from baseline at 6-months was − 0.4 ± 1.0% for the empagliflozin and − 0.3 ± 0.9% for the placebo groups, with adjusted between groups difference; − 0.2% (95% CI − 0.5 to 0.2%, P = 0.41). There were no differences between the groups for change in LV volumes, LVEF, and NT-proBNP from baseline to 6-month follow-up. There was also no between-group difference in terms of change in diastolic function, as measured by E/e’ ratio and indexed LA volume by echocardiography [11], and diastolic peak filling rate and time to peak filling rate by CMR [12].

**Table 1:** Baseline demographics, clinical and medication history of the participants

	Empagliflozin (10 mg) *n* = 44	Placebo *n* = 46
Men, n (%)	39 (89%)	44 (96%)
Age, years	64 (56–69)	65 (56–72)
Body mass index, kg/m^2^	27.1 (24.4–30.1)	26.5 (24.1–29.3)
Systolic blood pressure, mmHg	130 (120–147)	135 (126–147)
Diastolic blood pressure, mmHg	75 (69–82)	76 (71–81)
Heart rate, bpm	66 (60–75)	67 (60–74)
HbA1c, %	7.9 (7.5–8.4)	7.9 (7.3–8.7)
NT-proBNP, pg/mL	101 (43–197)	115 (59–217)
Cardiovascular history		
Hypertension, n (%)	40 (90.9%)	41 (89.1%)
Hypercholesterolemia, n (%)	32 (72.7%)	33 (71.7%)
TIA/Stroke, n (%)	8 (18.2%)	6 (13.0%)
History of PCI, n (%)	26 (59.1%)	18 (39.1%)
History of CABG, n (%)	24 (54.5%)	26 (56.5%)
Medication history		
Metformin, n (%)	43 (97.7%)	42 (91.3%)
Insulin, n (%)	11 (25%)	11 (23.9%)
ACEi / ARB, n (%)	37 (84.1%)	39 (84.8%)
Diuretic, n (%)	2 (4.5%)	5 (10.9%)
Cardiac MRI data		
Left ventricular mass index, g/m^2^	59.2 ± 10.7	62.1 ± 12.9
Left ventricular end diastolic volume index, mL/m^2^	62.9 ± 15.4	71.4 ± 15.6
Left ventricular end systolic volume index, mL/m^2^	26.7 ± 9.9	32.4 ± 11.9
Left ventricular ejection fraction, %	58.4 ± 7.0	55.3 ± 8.8
Regional wall motion abnormality, n (%)	17 (38.6%)	24 (52.2%)
Left ventricular peak filling rate, mL/sec	296.3 ± 99.4	294.5 ± 93.0
Time to peak filling rate (msec)	157.4 ± 46.3	165.9 ± 64.5
Echocardiographic data		
Average E/e′ ratio	10.8 ± 3.0	10.2 ± 3.1

Data shown in median (interquartile range), unless otherwise stated

*HbA1c* Hemoglobin A1c, *NT-proBNP* N-terminal pro-B-type natriuretic peptide, *TIA* Transient ischemic attack, *PCI* Percutaneous coronary intervention, *CABG* Coronary artery bypass grafting, *ACEi* Angiotensin-converting enzyme inhibitors, *ARB* Angiotensin receptor blockers

**Fig. 2:**
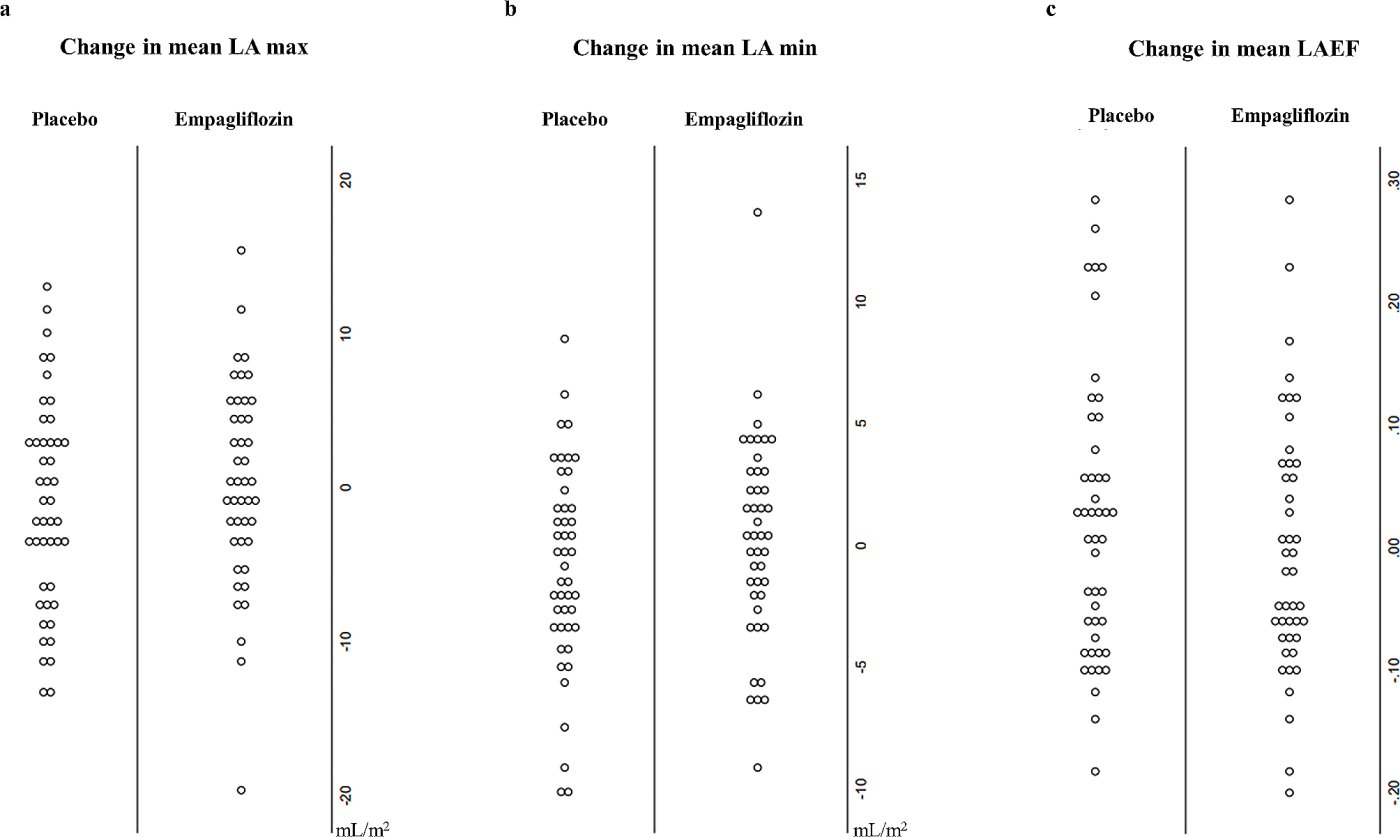
Six-month mean changes in **a** maximum left atrial volume, **b** minimum left atrial volume, and **c** left atrial total emptying fraction following treatment with empagliflozin versus placebo

CMR measures of LA volume and function at the baseline and 6-month visits are summarized in Table 2. There was no significant difference in baseline or 6-month indexed LAmax or LAmin, between the placebo and empagliflozin groups (Fig. 2). The between-group adjusted mean difference (empagliflozin minus placebo) was 0.99 mL/m^2^ (95% CI – 1.7 to 3.7 mL/m^2^; *p* = 0.47), and 0.87 mL/m^2^ (95% CI – 0.9 to 2.6 mL/m^2^; *p* = 0.32), for LAmax and LAmin, respectively. Additionally, there were no significant differences in adjusted mean difference in LAEF − 0.01 (95% CI – 0.05 to 0.03, *p* = 0.59), or components of active and passive LA function. There was no significant interaction between levels of NT-proBNP (≥ 125 pg/mL vs. < 125 pg/mL) on the between-group adjusted mean differences in LA indices (P = NS for all). There were significant interactions between presence of regional wall motion abnormality at baseline and treatment assignment for LAmax (*p* = 0.01), LAmin (*p* = 0.002), and LAEF (*p* = 0.01) at 6 months. The benefit of empagliflozin was more pronounced in those with regional wall motion abnormality at baseline, with smaller LA volumes and higher LAEF at 6 months. Intraclass correlation coefficients demonstrated excellent reliability (> 0.90) in the measurements of LAmax, LAmin, LApreA, LAAEF, and LAEF, and modest reliability (0.66) for LAPEF.

**Table 2:** CMR left atrial indices at baseline and at 6 months

CMR parameters	Empagliflozin (*n* = 44)			Placebo (*n* = 46)			Adjusted Difference Between Groups	95% CI	P value
	Baseline	6-month	Change in mean	Baseline	6-month	Change in mean			
LAmax, mL/m^2^	26.4 ± 8.4	26.7 ± 9.2	0.3 ± 6.5	28.7 ± 8.2	27.5 ± 9.1	– 1.2 ± 6.7	0.99	– 1.7 to 3.7	0.47
LAmin, mL/m^2^	11.1 ± 5.7	11.4 ± 6.3	0.3 ± 4.1	12.6 ± 5.0	11.8 ± 5.9	– 0.8 ± 4.1	0.87	– 0.9 to 2.6	0.32
LAPEF	0.27 ± 0.09	0.26 ± 0.09	0.00 ± 0.11	0.26 ± 0.08	0.26 ± 0.10	0.00 ± 0.11	0.01	– 0.03 to 0.05	0.65
LAAEF	0.44 ± 0.14	0.44 ± 0.12	– 0.006 ± 0.12	0.42 ± 0.09	0.45 ± 0.12	0.03 ± 0.12	– 0.02	– 0.07 to 0.02	0.34
LAEF	0.59 ± 0.12	0.59 ± 0.11	0.00 ± 0.10	0.56 ± 0.10	0.58 ± 0.12	0.02 ± 0.11	– 0.01	– 0.05 to 0.03	0.59
pPER, mL/sec	– 124.8 ± 49.7	– 118.9 ± 52.7	5.9 ± 43.7	– 123.0 ± 46.4	– 105.5 ± 54.0	17.5 ± 59.2	– 12.4	– 32.3 to 7.4	0.22
pAER, mL/sec	– 175.4 ± 57.5	– 197.9 ± 66.8	– 23.6 ± 81.3	– 183.4 ± 75.5	– 198.5 ± 85.8	– 13.4 ± 92.1	– 3.6	– 35.1 to 27.8	0.82

Data expressed as mean ± standard deviation and analyzed using ANCOVA adjusting for baseline values

*CMR* Cardiac magnetic resonance imaging, *CI* Confidence Interval, *LAmax* Maximum LA volume, *LAmin* Minimum LA volume, *LAPEF* Left atrial passive emptying fraction, *LAAEF* Left atrial active emptying fraction, *LAEF* Left atrial total emptying fraction, *pPER* Peak passive emptying rate, *pAER* Peak active emptying rate

The relationships between LA parameters and select LV and clinical parameters are demonstrated in Table 3. There was no significant correlation between LVMi and changes in LA size or function from baseline to 6 months. However, there was a modest significant correlation between changes in LV and LA volumes. There was also a significant correlation between LV peak filling rate and active, as well as total LA emptying fraction. There was a negative correlation between changes in NT-pro-BNP and active, as well as total LA emptying fraction.

**Table 3:** Correlation coefficients (rho) between changes in left atrial and left ventricular indices over 6 months

	Δ LAmax	Δ LAmin	Δ LAPEF	Δ LAAEF	Δ LAEF	Δ pPER	Δ pAER
Δ LVMI, g/m^2^	– 0.14	– 0.092	0.024	– 0.025	– 0.034	0.12	– 0.075
Δ LVEDVI, mL/m^2^	0.42**	0.27*	0.11	– 0.054	0.072	– 0.29**	– 0.098
Δ LVESVI, mL/m^2^	0.22*	0.15	0.066	– 0.042	0.020	– 0.19	0.024
Δ LVEF, %	0.071	0.026	0.017	0.042	0.061	0.072	– 0.19
Δ Peak filling rate, (ml/sec)	0.001	– 0.17	0.061	0.23*	0.26*	– 0.13	– 0.064
Δ Time to peak filling rate (msec)	0.042	0.073	0.089	– 0.14	– 0.072	0.009	0.093
Δ Average E/e′ ratio	0.16	0.18	0.004	– 0.098	– 0.084	0.078	– 0.006
Δ NT-proBNP, pg/mL	0.18	0.26*	– 0.12	– 0.29**	– 0.24*	– 0.072	– 0.079
Δ HbA1c, %	– 0.12	– 0.041	– 0.066	0.070	0.048	– 0.16	– 0.005
Δ Systolic blood pressure, mmHg	0.089	0.20	– 0.18	– 0.15	– 0.28**	– 0.062	0.090
Δ Diastolic blood pressure, mmHg	0.049	0.13	– 0.17	– 0.071	– 0.20	– 0.12	0.006

*LVMI* Left ventricular mass index, *LVEDVI* Left ventricular end-diastolic volume index, *LVESVI* Left ventricular end-systolic volume index, *LVEF* Left ventricular ejection fraction, *NT-proBNP* N-terminal pro b-type natriuretic peptide, *HbA1c* Hemoglobin A1c

*P < 0.05, **P < 0.01

## Discussion

In this post-hoc sub-study of the EMPA-HEART CardioLink-6 trial, we investigated the effect of empagliflozin in addition to standard of care in patients with T2DM and CAD. Our findings demonstrate that SGLT2 inhibition with empagliflozin for 6 months did not have an impact on LA volume and function, as measured by CMR.

SGLT2 inhibitors, beyond glucose-lowering effects, have important cardio-renal benefits, with both direct and indirect mechanisms of action [4]. Studies which have directly measured the impact of SGLT2 inhibitors on cardiac structure and function have demonstrated improvements in LV remodelling and LV mass regression in patients with heart failure [13]. In EMPA-HEART CardioLink-6 [5] and DAPA-LVH (Does Dapagliflozin Regress Left Ventricular Hypertrophy In Patients With Type 2 Diabetes) [14], SGLT2 inhibition led to LV mass regression in patients with T2DM and CAD or T2DM and LV hypertrophy, respectively. In subsequent sub-studies of EMPA-HEART CardioLink-6, our group showed that empagliflozin led to reduction in extracellular fibrosis [6], but had no impact on measures of LV diastolic function, as measured by echocardiography [11] or CMR [12]. This contrasts with the results from other groups, demonstrating improvements in diastolic function. Rau et al. reported that in 42 patients with diabetes, treatment with empagliflozin did not impact LV systolic function, but did improve diastolic parameters, as measured by E/eʹ [15]. This difference was apparent at day 1 of treatment and was sustained throughout the study duration of 3 months. In the EmDia (Effects of Empagliflozin on Left Ventricular Diastolic Function Compared to Usual Care in Type 2 Diabetics) trial, participants with T2DM and elevated E/eʹ were randomized to empagliflozin versus placebo. At three months, empagliflozin resulted in improved diastolic function (lower E/e´ ratio), which was consistent in both patients with or without HFpEF [16].

LA size and function have been shown to predict the development of adverse cardiovascular outcomes in patients with atrial fibrillation, ischemic heart disease, and LV dysfunction [9]. Notably, adverse LA remodelling is intricately linked to the pathophysiology of HF, impacting atrial contractile function, and resulting in atrial fibrosis and electrophysiological remodelling [17]. In the general population, changes in LA volume parallel changes in blood pressure, LV mass, NT-proBNP, and visceral fat mass [18]. LA size and function, therefore, have emerged as surrogate markers of interest, particularly in patients at risk of or who have subclinical disease. In the PARABLE (Personalized Prospective Comparison of ARNI With ARB in Patients With Natriuretic Peptide Elevation) study, for instance, investigators randomized asymptomatic participants with hypertension or T2DM, elevated natriuretic peptide and dilated LA volume by echocardiography to sacubitril/valsartan or valsartan alone. Unexpectedly, sacubitril/valsartan resulted in larger maximal LA volume, despite greater reduction in pulse pressure and NT-proBNP [19].

Several studies have examined the impact of SGLT2 inhibitors on atrial function. In an animal model of obese rats with metabolic syndrome and HFpEF, Bode et al. demonstrated that the SGLT2 inhibitor sotagliflozin improved echocardiographic measured LA volume. This improvement paralleled decreased in-vitro LA cellular arrhythmogenesis [20]. In humans, DAPA-MODA (Impact of Dapagliflozin on Cardiac Remodelling in Patients with Chronic Heart Failure) enrolled 162 participants with stable, chronic HF, where approximately half had preserved ejection fraction. LA volume, as measured by echocardiography, was significantly lowered by the SGLT2 inhibitor dapagliflozin, which also led to a reduction in LV mass, volumes, and NT-proBNP [21]. In SUGAR-DM-HF (Studies of Empagliflozin and Its Cardiovascular, Renal and Metabolic Effects in Patients With Diabetes Mellitus, or Prediabetes, and Heart Failure), 105 participants with HF (LVEF ≤ 40%) and diabetes or prediabetes, were randomized to empagliflozin or placebo. Using CMR to ascertain the endpoints, empagliflozin led to a reduction in LV end diastolic volume, but did not impact LV mass or LA volumes [22]. In a sub-study of Empire HF (Empagliflozin in Heart Failure Patients with Reduced Ejection Fraction), which also randomized patients with HF (LVEF ≤ 40%), empagliflozin reduced both LV and atrial volumes [23].

In participants without HF, the results have been mostly neutral. Aslan et al. studied 62 individuals with T2DM. After six months, there were no significant changes in LA volumes, total emptying and passive emptying volume, with a very small difference in active emptying volume [24]. Ersboll et al. reported that in patients with T2DM and established CVD or CV risk factors, treatment with empagliflozin did not lead to a significant change in LA volume, despite improvement of LV mass and diastolic function measured by echocardiography [25]. In IDDIA (Impact of Dapagliflozin on Left Ventricular Diastolic Dysfunction in Patients with Type 2 Diabetes Mellitus), Shim et al. enrolled 60 participants with diabetes and diastolic dysfunction on echocardiogram [26]. At 24 weeks, participants assigned to dapagliflozin had improvement in their diastolic function during stress echocardiography, but there was no difference in LA volumes. A recent study using LA strain analysis, however, suggests that empagliflozin ameliorates both LA reservoir and LA contractile function in patients with T2DM [27].

To our knowledge, our study is the first double blinded RCT using CMR to evaluate the effect of empagliflozin on LA volume and function in patients with T2DM and CAD. Our study expands upon the previous literature and highlights that in this population with mild diastolic dysfunction and normal LA volumes [28], SGLT2 inhibition with empagliflozin did not significantly impact LA size or function at six months. The results of this analysis support previous findings suggesting that SGLT2 inhibition does not impact LA volume, in the absence of heart failure, a finding which is congruent with the lack of change in NT-proBNP in the study. In our study, the levels of NT-proBNP did not modify the effects of empagliflozin upon LA size or function at six months. There was a significant interaction between treatment assignment and the presence of regional wall motion abnormality at baseline, where the beneficial effect of empagliflozin on LA volume and function was more pronounced. However, this finding should be seen as hypothesis generating, given its post-hoc nature and the limited sample size.

There are limitations to our study. The sample size was relatively small, and powered to detect differences in LV mass rather than LA parameters. As such our study may be subject to type II error in post hoc analyses. Furthermore, the 6 month duration may not have been long enough for us to observe measurable LA remodelling. However, most previous studies on the impact of SGLT2 inhibition on cardiac structure and function followed similar timeframes [13]. There are also important strengths to our study. All participants were enrolled at one center, with dedicated CMR equipment, which significantly reduced variations due to protocols or technical deviations. All CMR measurements were performed by an experienced operator, blinded to patients’ treatment group or clinical information.

In conclusion, in patients with T2DM and CAD, but without prevalent HF, SGLT2 inhibition with empagliflozin for 6 months did not significantly impact LA volumes and function. Future studies with longer follow-up duration, should be performed to better elucidate the interplay between LA and LV diastolic function.

## Data Availability

The datasets analyzed during the current study are not publicly available but are available from the corresponding author on reasonable request.
